# XBB.1.5: A new threatening SARS-CoV-2 Omicron subvariant

**DOI:** 10.3389/fmicb.2023.1154296

**Published:** 2023-04-17

**Authors:** Md. Aminul Islam, Fatema Hasan Kaifa, Deepak Chandran, Manojit Bhattacharya, Chiranjib Chakraborty, Prosun Bhattacharya, Kuldeep Dhama

**Affiliations:** ^1^Advanced Molecular Lab, Department of Microbiology, President Abdul Hamid Medical College, Kishoreganj, Bangladesh; ^2^COVID-19 Diagnostic Lab, Department of Microbiology, Noakhali Science and Technology University, Noakhali, Bangladesh; ^3^Department of Veterinary Sciences and Animal Husbandry, Amrita School of Agricultural Sciences, Amrita Vishwa Vidyapeetham University, Coimbatore, Tamil Nadu, India; ^4^Department of Zoology, Fakir Mohan University, VyasaVihar, Balasore, Odisha, India; ^5^Department of Biotechnology, School of Life Science and Biotechnology, Adamas University, Kolkata, West Bengal, India; ^6^COVID-19 Research @KTH, Department of Sustainable Development, Environmental Science and Engineering, KTH Royal Institute of Technology, Stockholm, Sweden; ^7^Division of Pathology, ICAR-Indian Veterinary Research Institute, Bareilly, Uttar Pradesh, India

**Keywords:** COVID-19, Omicron, XBB.1.5 sub-lineage, SARS-CoV-2, new variants, transmission

## Introduction

COVID-19 pandemic has caused over 6.71 million deaths out of 660 million confirmed cases as of January 12, 2023 (Rakib et al., 2021, 2022; WHO, 2022). SARS-CoV-2, the virus responsible for this worldwide pandemic is a member of the broad family of viruses known as coronaviruses (CoVs) (Islam et al., 2021, 2022a,b). It seems difficult to terminate the present pandemic despite continued vaccination programs and booster shots since new SARS-CoV-2 variants and subvariants are constantly emerging with different potentials of transmissibility, infectivity, and fatality (Jakariya et al., 2021; Sakib et al., 2021; Wong, 2022). Evaluating novel variants based on epidemic intelligence along with screening for genomic variants is regularly done on behalf of (ECDC, 2023). Omicron (B.1.1.529) and its subvariants BA.1, BA.2, BA.3, BA.4, BA.5, and its descendant lineages are the only variants of concerns (VOCs) currently circulating, while Alpha (B.1.1.7), Beta (B.1.351), Gamma (P.1), and Delta (B.1.617.2) are now categorized as previously circulating VOCs (Dhama et al., 2022). Unexpectedly, the Delta variant caused a sharp rise in COVID-19 cases and more fatalities in 2021, while Omicron variant then caused a gigantic and absolutely massive rise in 2022. The Omicron variant (Pango lineage B.1.1.529) has spawned numerous offshoots, including the BA.2, BA.3, BA.1, and BA.1.1, BA.2.12.1, BA.2.75, BA.2.75.2, BA.4, BA.4.6, and BA.5, BQ.1, BQ.1.1 (BA.4/5), BF.7, and XBB.1.5 (Fernandes et al., 2022). The BA.2, BA.4, and BA.5 have been discovered in 2022; almost all of the variants have South African origin except for BA.2.75 which was discovered in India (Dhama et al., 2023). BQ.1.1 now makes up 34% of cases nationwide, whereas XBB.1.5 makes up 28% of instances. In Nebraska, BA.5 and BQ.1 accounted for 23% of cases each as of December 31, 2022. Other Omicron variations may include XBB and XBB.1.1, yet, they are not specifically included in the most recent study, following the recent upheaval of BQ.1.1, accounting 23% of cases based in Nebraska (Nebraska Medicine, 2023). The most recent information also suggests the appearance of the variant XBB.1.5, which is a recombinant of the subvariants BA.2.10.1 and BA.2.75. Omicron XBB1.5 sub-lineage has been listed by ECDC as a variant of interest (VOI) as of January 12, 2023 (ECDC, 2023). According to ECDC arithmetical model in the EU/EEA, the XBB1.5 sub lineage of XBB, SARS-CoV-2 Omicron variant could turn into dominant after 1 to 2 months where it is identified 2.5% last 2 weeks of 2022 and spreading quickly (12% detected) in the United States (ECDC, 2023a).

Like other variants, the Omicron variant is composed of a variety of lineages and sublineages. Although these lineages are extremely analogous to one another, variations across lineages may influence how the virus behaves. The XBB strain is a hybrid of BA.2.10.1 and BA.2.75 varieties. Initial research suggests that XBB has a greater risk of reinfection than the other circulating Omicron sublineages (Uraki et al., 2023). Only those who contracted XBB prior to the emergence of Omicron type were at risk of reinfection. The hypothesis that other Omicron lineages could be able to evade the immune responses they have already caused is, as of now, unproven (WHO, 2023a). The rapid spread of the XBB.1.5 Omicron subvariant in the northeastern United States has prompted concern from the World Health Organization (WHO). Until now, the transmission rate of this variant has been the highest of any subtype (WHO, 2023b). XBB.1.5 shares the same resistance to vaccination and disease-induced immune reactivity as its close siblings XBB and XBB.1. XBB.1.5, however, contains a mutation that increases its ability to adhere to cells, providing it a competitive advantage. When a virus is able to infect people who have been previously exposed to it, either by infection or immunization, it is said to exhibit immune evasiveness. The XBB.1.5 strain achieved this property by creating a novel mutation in its receptor-binding domain (RBD) termed F486P. The extent to which it causes or contributes to more severe disorders is unclear. According to the experts, this is an extremely remote possibility (Kurhade et al., 2022).

The SARS-CoV-2 subvariants BQ.1.1 and XBB.1 have spread widely because they are more successful at growing than the vast majority of Omicron mutants. However, recent studies have shown that the recombinant mutant XBB subvariant XBB.1.5 has a substantial growth advantage over BQ.1.1 and XBB.1 (Tamura et al., 2022). Because of its greater transmissibility, XBB.1.5 has swiftly become the predominant strain in the United States and is almost certainly may be the cause of rising cases in the forthcoming time amid the ongoing COVID-19 pandemic. XBB/XBB.1 has been demonstrated to be even more resistant to neutralization by plasma/serum from vaccinated or recovering healthy individuals than BQ.1.1. Ser486Pro is an unusual 2-nucleotide change on the spike (S) protein that is only seen in XBB.1.5 and not in XBB.1. Urgent research into the causes of XBB.1.5's rapid propagation, especially the part played by Ser486Pro, is required (Cao et al., 2022; Yue et al., 2023).

This winter, most respiratory viruses have been circulating at higher rates than usual. To date, XBB.1.5 has been found to be the Omicron sub-variant that spreads the most rapidly. Its rapid proliferation can be attributed to the additional mutations it carries. The cause is a mutation in a region of the spike protein required for binding to its receptor, ACE2 (Angiotensin Converting Enzyme 2). Because of its superior ability to invade human cells, this variety has emerged possibility as the most dangerous. In many simulations, XBB.1.5 was found to have a higher transmission R value and infection rate than earlier versions. The Centers for Disease Control and Prevention (CDC) reports that only 15% of Americans age 5 and older have received a bivalent booster, suggesting that many people are currently less protected than they may be against the new strain (CDC, 2023). Certain data suggest that the probability of acquiring a chronic case of COVID-19 is lower for people who contracted an earlier form of the Omicron variations as compared to those who got the Delta strain. But if XBB.1.5 spreads, even a small number of people with chronic issues could substantially increase chronic COVID cases. Regardless of its current status, the CDC predicts an increase in reported infections over the next few weeks. The fact that this variant has recently caused an uptick in hospitalization rates in New York City suggests that it has a propensity to do so in other areas where it is prevalent. Breathing problems, headaches, sore throats, blocked noses, achy bodies, and fevers are all symptoms frequently reported by those with the Omicron XBB sub-variant lineage. At least 74 nations and 43 US states have confirmed cases of XBB, according to the https://outbreak.info/. Taking into account sample collection dates up to and including October 22, 2022, the novel variant XBB.1.5 was first identified in the United States at that time (ECDC, 2023). The CDC claims that the origin of the novel XBB.1.5 variant is unknown, but that outbreaks have been predominantly connected to North-Eastern nations, as is evident from Table 1.

**Table 1:** The outbreaks of novel XBB1.5 variant since October 2022.

**Country where the variant has been detected**	**Date of first detection**	**Places of emergence**	**Number of cases by late December to early January**	**References**
USA	22nd October	New York	40%	(ECDC, 2023)
France	2nd January		15 cases	(Nebraska Medicine, 2023)
Germany	Early Januray	Brandenburg	Not disclosed	(New work times, 2023)
Canada	4th January	Alberta	1 case	(UK Daily News, 2023)
Australia	3rd January	New South Wales	8 cases	(ECDC, 2023a)
India	Early Januray	Gujarat and Karnataka and Rajasthan	5 cases	(ECDC, 2023a)
New Zealand	1st week of Januray		2 cases	(ECDC, 2023a)
Fiji Island	Late 1st week of Januray	Fiji's border	Not disclosed	(ECDC, 2023a)

According to the CDC, the younger version, known as XBB.1.5, accounted for 27.6% of cases nationwide and 72.0% of new cases in the Northeast, indicated precisely in the above table (CDC, COVID-19).

According to Dr. Lynora Saxinger, an expert in infectious diseases at the University of Alberta (NB News, 2023), the new subvariant appears to be “an antibody-escaper,” because of its enhanced proliferation and mutational powers. Recent laboratory investigations reveal that XBB can hide from antibodies associated with prior COVID-19 infections or vaccines (ECDC, 2023b), suggesting that an exposed person is more likely to become unwell, become reinfected, and develop symptoms. Scientist and assistant professor at Peking University Yunlong Cao claims that XBB.1.5 is superior to the XBB.1 form at attaching to cells *via* a critical receptor and at evading protective antibodies. Its rapid spread, especially in the United States, is very concerning, despite the lack of evidence that it causes more severe illness than other Omicron variants.

Due to their ability to evade the immune system, causing vaccine breakthrough infections and reinfection, the emerging newer Omicron strains indicate that masks are still necessary and it is not yet safe to relax. Vaccines and booster doses can reduce hospitalizations, disease severity, deaths, and long-term effects of SARS-CoV-2 variants including Omicron and its subvariants (Ahmed et al., 2021). The best way to protect yourself from SARS-CoV-2 and its variations is to vaccinate (Zhou et al., 2022). Omicron can be treated, deaths avoided, and major issues get resolved with the use of booster doses and immunizations. Against the Omicron form, even three doses of the COVID-19 vaccine only offer limited protection, therefore additional boosters are required to confer better immunity. There is a pressing need for improved vaccination methods and technologies as well as universal access to immunizations. When Omicron subvariants increase in number, newer and better vaccines with higher efficacy and mAbs with high potential are required in the clinics for treating COVID-19 patients (Fernandes et al., 2022). Vaccines that can be administered by the nose (intranasal, mucosal vaccines), nanoparticle-based vaccines, universal vaccines aimed at pan-beta-coronaviruses, and mutation proof vaccines are urgently required at this time (Akkiz, 2022; Ke et al., 2022) An increase in surveillance and monitoring, strict vigilance, the adoption of suggested COVID-19 protection and control strategies, and increased immunization programs and booster doses are all urgently needed to combat emerging SARS-CoV-2 variants and subvariants (Ahemd et al., 2021). Limiting the transmission and spread of COVID-19 and its associated mortality and the emergence of new Omicron subvariants can be achieved through the use of face masks, frequent hand washing, social/physical distancing, adequate cleaning and disinfection measures, and the avoidance of crowded settings and mass gatherings (Aleem et al., 2022).

Patients frequently take natural drugs that inhibit the S protein (iguesterin, baicalin), 3CLpro (iguesterin, cryptotanshinone), helicase (silvestrol), and RdRp. Among these are *Allium sativum, Camellia sinensis, Zingiber officinale, Nigella sativa*, and several Echinacea species (Sharun et al., 2022). Although terpenoids have been shown to be useful in suppressing viral replication, there are also alkaloids with strong anti-coronavirus activities. Patients with severe cases of COVID-19 typically have severely weakened innate immunity. Several antiviral medications, such as Monalizumab, interferon, and chloroquine, can restore the function of CD8+ T and NK cells (generally decreased in severe cases). However, the best way to ensure your health, both now and in the future, is to avoid getting sick in the first place. In an effort to keep everyone healthy, additional measures need to follow boost immunity, such as getting enough sleep, regulating stress, reducing inflammation, improving diet, taking healthy nutritious foods, probiotics, staying hydrated, and exercising. Rapid, accurate, timely diagnosis of COVID-19, SARS-CoV-2 whole genome sequencing, booster doses vaccines, mask, proper hygiene rules, one health guidelines, and wastewater based surveillance to monitor hotspots might follow to tackle this new XBB.1.5.

## Author contributions

MI: conceptualized, wrote the first draft, and edited. FK, DC, CC, KD, and PB: updated the manuscript, edited, and reviewed. All authors have critically reviewed and approved the final draft.
